# Association Between Atherosclerosis-Related Cardiovascular Disease and Uveitis: A Systematic Review and Meta-Analysis

**DOI:** 10.3390/diagnostics12123178

**Published:** 2022-12-15

**Authors:** Xinyi Gao, Tonglian Lv, Guangping Li, Gary Tse, Tong Liu

**Affiliations:** 1 Tianjin Key Laboratory of Ionic-Molecular Function of Cardiovascular Disease, Department of Cardiology, Tianjin Institute of Cardiology, Second Hospital of Tianjin Medical University, Tianjin 300211, China; 2 Kent and Medway Medical School, Canterbury CT2 7NZ, UK

**Keywords:** uveitis, ankylosing spondylitis, atherosclerosis, cardiovascular risk, intima-media thickness, carotid plaques

## Abstract

Background: Uveitis is not only an intraocular inflammatory disease, but also an indicator of systemic inflammation. It is unclear whether uveitis can increase the risk of cardiovascular disease (CVD) through the atherosclerotic pathway. Methods: PubMed and Embase databases were searched until 5 September, 2022. Original studies investigating uveitis and cardiovascular events were selected. The random-effects model was used to calculate the difference of groups in pooled estimates. Results: A total of six observational studies that included mainly ankylosing spondylitis (AS) patients were included. Of these, three studies reported data on carotid plaques and carotid intima-media thickness (cIMT) and the other three studies provided data on atherosclerosis-related CVD. No significant difference was found in cIMT between uveitis and controls (MD = 0.01, 95% CI = −0.03–0.04, *p* = 0.66), consistent with the findings of carotid plaque incidence (OR = 1.30, 95% CI = 0.71–2.41, *p* = 0.39). However, uveitis was associated with a 1.49-fold increase in atherosclerosis-related CVD (HR = 1.49, 95% CI = 1.20–1.84, *p* = 0.0002). Conclusions: Uveitis is a predictor of atherosclerosis-related CVD in AS patients. For autoimmune disease patients with uveitis, earlier screening of cardiovascular risk factors and the implementation of corresponding prevention strategies may be associated with a better prognosis.

## 1. Introduction

Uveitis is the inflammation of the middle layer of tissue that lies between the inner retina and the outer fibrous layer of the eye. Depending on the anatomical location involved in inflammation, uveitis can be categorized as anterior, intermediate, posterior or panuveitis. By duration, uveitis lasting less than and more than three months is defined as acute or chronic, respectively. As an intraocular inflammation disease, uveitis is the leading cause of visual impairment and is recognized as the fifth or sixth major cause of blindness worldwide [1,2]. Unlike other diseases that predispose blindness, there is no obvious age tendency in uveitis patients. All age groups, including children, can be affected.

In accordance with the currently accepted etiological classifications, uveitis is considered to be primarily of autoimmune or immune-mediated origin, with the exception of infectious uveitis which is due to infection and/or trauma. The central focus of this review is autoimmune uveitis, a non-infectious inflammatory process within the eye, commonly idiopathic, i.e., isolated from an autoimmune disease (idiopathic autoimmune uveitis: I-AU), whereas less than 20% of cases are associated with systemic disease which is believed to be systemic disease-associated autoimmune uveitis: SDA-AU [3,4]. It is well known that a variety of systemic autoimmune disorders are associated with uveitis, including Behçet’s disease, psoriasis, spondylarthritis and so on. Uveitis is reported to be involved in 25 to 30% of patients with psoriatic arthritis [5], approximately 33% with ankylosing spondylitis [6] and 30 to 70% of patients with Behçet’s disease [7]. The occurrence of uveitis due to ocular involvement may precede or follow the onset of systemic symptoms, depending on the autoimmune disease. Additionally, the onset of symptoms and the effected sites of uveitis vary according to different disorders. As for the pathogenesis of autoimmune uveitis, limited to the vulnerability of ocular tissues, existing studies have not fully elucidated this. However, the development of autoimmune uveitis is still considered to be an activated abnormal T cell-mediated immune response with B cell involvement, triggered by inflammation targeting retinal or cross-reactive antigens [8]. With a gradual deepening of our understanding, uveitis is now recognized not only as an intraocular inflammation, but also as an indicator of systemic inflammation. Previous studies have demonstrated that in addition to aqueous and vitreous humor, increased pro-inflammatory cytokines and chemokines have also been detected in the tears and serum samples of patients with uveitis [9,10]. Pro-inflammatory cytokines and chemokines have been shown to exert an essential role in the pathogenesis of uveitis [11,12,13,14].

Atherosclerosis is the most common pathological basis of cardiovascular and cerebrovascular diseases [15], which is known to be closely associated with the inflammatory response [16,17]. Notably, emerging evidence from animal studies and clinical interventions has indicated that autoimmune responses are involved in the occurrence and development of atherosclerosis [18,19,20]. Thus, it is not complicated to know that systemic inflammation and autoimmune responses can increase the risk of cardiovascular disease through the atherosclerotic pathway. The relationship between atherosclerosis and inflammatory processes has been revealed in patients with rheumatoid arthritis [21,22], Behçet’s disease [23] and systemic lupus erythematosus [24].

As an intraocular inflammation, uveitis can cause blindness, but as an indicator of systemic inflammation, its separate effect on other systems remains unclear. In the cardiovascular system, the prolonged presence of uveitis as a chronic inflammatory condition can promote the inflammatory cytokine cascade that activates circulating cytokines and secondary adhesion molecules enabling further endothelial damage, facilitating the formation of atherosclerosis and increasing the risk of atherosclerosis-related cardiovascular disease (CVD) [20,25]. Whereas studies on the link between uveitis and atherosclerosis-related CVD are still lacking. Hence, we performed a systematic review and meta-analysis aiming to explore the association between uveitis and atherosclerosis-related CVD.

## 2. Methods

The current systematic review and meta-analysis was conducted in accordance with the preferred reporting information for systematic reviews and meta-analysis (PRISMA) recommendations [26].

### 2.1. Search Strategy

Two investigators conducted a systematic search of the PubMed and EMBASE databases for all original articles related to uveitis and cardiovascular events from the inception of the databases till 5 September 2022. The combination of terms ‘uveitis’ and ‘cardiovascular’ were used for the literature search with no language or sample size restrictions. In addition, possible missing studies were manually searched and reviewed in the reference lists of the relevant articles.

### 2.2. Study Selection

To investigate the association between uveitis and cardiovascular events, the following criteria were applied for study inclusion: (1) published in English; (2) uveitis at baseline as a predictor; (3) reporting cardiovascular-related events (e.g., hypertension, heart failure, coronary disease, structural heart disease, cardiac arrhythmias, etc.) as outcome measures; (4) providing an odds ratio (OR), risk ratio (RR) or hazard ratio (HR) with corresponding 95% confidence intervals (CI) or other computable data for cardiovascular-related event outcomes in patients with uveitis versus those without; (5) observational studies included prospective cohort studies, retrospective cohort studies, case–control studies or cross-sectional studies as study designs. To expand the number of studies, conference abstracts that provided valuable data were also included. The reviews, case reports, letters, editorials, comments and articles not related to the research topic were excluded from the list of articles. Two investigators first independently screened the title and abstracts of the literature to exclude those not associated with the study topic, and then performed a full-text analysis to screen the studies for eligibility. Any uncertainties or discrepancies between the two reviewers were resolved through discussion and independent assessment by a senior reviewer to reach a consensus.

### 2.3. Data Extraction and Study Quality Assessment

Data extraction was performed by two non-blinded authors separately, using a predefined standard data correlation extraction table, and the extracted results were subsequently cross-checked to ensure divergence-free data collection. The following information was extracted from each study: first author, year of publication, study design, origin of study, study population, sample size, gender distribution, proportion of with and without uveitis in each study, definition of uveitis, type of uveitis, cardiovascular outcomes, duration of study, and follow-up.

Quality assessment of the cohort studies included was conducted using the Newcastle–Ottawa quality assessment scale in terms of three domains: selection, comparability and outcome. Studies were graded as high-quality (≥7 stars), moderate-quality (4–6 stars) and low-quality (≤3 stars) based on the final score. Quality assessment of the remaining three cross-sectional studies was conducted by utilizing the American Agency for Healthcare Research and Quality cross-sectional study evaluation criterion. Likewise, studies were categorized as high-quality if they met ≥8 criteria, moderate if they met 4–7 criteria, and poor if they met ≤3 criteria based on the scores of the 11 items. 

### 2.4. Statistical Analysis

For the three retrospective database-based cohort studies included, the inverse variance method was used to evaluate generic inverse variance in terms of the log of HR, standard error (SE) of the log of HR, and 95% CI of each trial. For the three studies on atherosclerosis included in the meta-analysis, OR with 95% CI were used as summary statistics for carotid plaques. Mean difference (MD) and 95% CI were used as an effect measure in the analysis of the continuous variable cIMT. Statistical heterogeneity in this study was quantified using the Cochran’s Q test, which was set at a significant level using the *p* value < 0.10. This test was supplemented by the I^2^ statistic, with an I^2^ value ranging from 0–25% indicating insignificant heterogeneity, 26–50% indicating low heterogeneity, 51–75% indicating medium heterogeneity, and 76–100% indicating high heterogeneity. The random effects model was chosen when the heterogeneity was significant, i.e., *p* value < 0.10 or I^2^ > 50%. Leave-one-out sensitive analysis and subgroup analysis were further conducted when significant heterogeneity was observed. Publication bias was evaluated by inspecting the funnel plots. Reported values were two-tailed, and *p* values < 0.05 were considered statistically significant for hypothesis testing. All the meta-analyses were conducted via Review Manger, version 5.4 (RevMan; The Cochrane Collaboration, Oxford, UK).

## 3. Results

### 3.1. Literature Search

**Figure 1:**
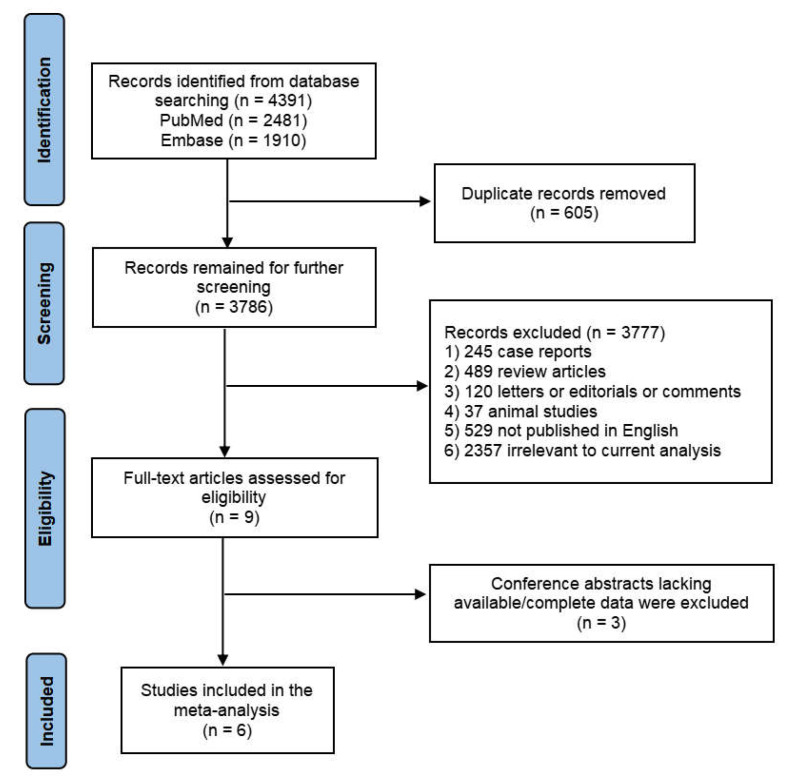
Flowchart of the study selection.

A flowchart detailing the entire process of the literature search and screening is illustrated in Figure 1. A total of 4391 potentially relevant publications was retrieved from PubMed and Embase, from which 605 duplicated records were excluded. Subsequently, through an initial perusal of titles and abstracts, 3777 records were excluded for the following reasons: 245 were case reports; 489 were review articles; 120 were letters, editorials, or comments; 37 were animal studies; 529 were not published in English and 2357 were irrelevant to our current topic. The remaining nine articles were then perused in full to assess their eligibility, and three of the conference abstracts were excluded due to lack of available/complete data. Finally, a total of six studies (five articles and one conference abstract) satisfied our inclusion criteria and were included in this meta-analysis.

### 3.2. Characteristics of the Included Studies

The main characteristics of the included studies and their quality scores are illustrated in Table 1. A total of six studies, three of which were retrospective database-based cohort studies reporting atherosclerosis-related CVD and the others three were cross-sectional studies reporting atherosclerosis. The sample sizes of the eligible studies varied from 125 to 17,720, and five studies were from populations with axial spondylarthritis (mainly AS) and one study was from children. The median or mean follow-up duration for the five studies that provided data was more than 3 years. Regarding the methodological quality assessment for the selected studies, all of the three cohort studies were rated as high-quality due to NOS scores ≥ 7; one of the three cross-sectional studies was rated as high-quality, one was rated as moderate-quality and the other was rated as low-quality based on AHRQ entries. A lower conference abstract score may be due to the limited data provided to allow for adequate assessment. The detailed results of quality assessment are shown in Tables S1 and S2.

Regarding cardiovascular-related outcomes, two cross-sectional studies involving a population with axial spondylarthritis showed no statistical difference in the incidence of carotid plaques between uveitis or non-uveitis groups. Three database-based cohort studies reporting atherosclerosis-related CVD involved AU patients, and two of which reported a positive association between AU and atherosclerosis-related CVD. In addition, these two cohorts included patients with posterior segment involving uveitis, and both reported that posterior segment involving uveitis positively correlated with atherosclerosis-related CVD (Table 2). Multivariate variables adjusted for estimates of HR are detailed in Table S3.

### 3.3. Association between Uveitis and Carotid Atherosclerosis

**Figure 2:**
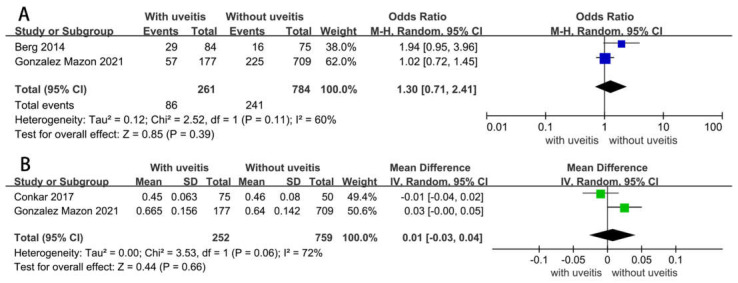
(**A**) Forest plots showing pooled OR with 95% CI of carotid plaques for patients with uveitis versus those without. (**B**) Forest plots showing pooled MD with 95% CI of cIMT for patients with uveitis versus those without.

The effects of uveitis on carotid atherosclerosis are summarized in Figure 2. A total of two studies providing data on the association of uveitis with carotid plaques were eligible for analysis. Subjects with uveitis versus those without were found to possess no significant difference in the incidence of carotid plaque formation (OR = 1.30, 95% CI = 0.71–2.41, *p* = 0.39) (Figure 2A). A significant heterogeneity was observed between the two studies (I^2^ = 60%, *p* = 0.11). A total of two studies investigated the effects of uveitis on subclinical atherosclerosis by pooling data from cIMT under ultrasound. Similarly, no significant association was detected between uveitis and cIMT (MD = 0.01, 95% CI = −0.03–0.04, *p* = 0.66), with a high heterogeneity (I^2^ = 72%, *p* = 0.06) (Figure 2B). Visual inspection of funnel plots shows little publication bias (Figure S2).

### 3.4. Association between Uveitis and Atherosclerosis-Related CVD

**Figure 3:**
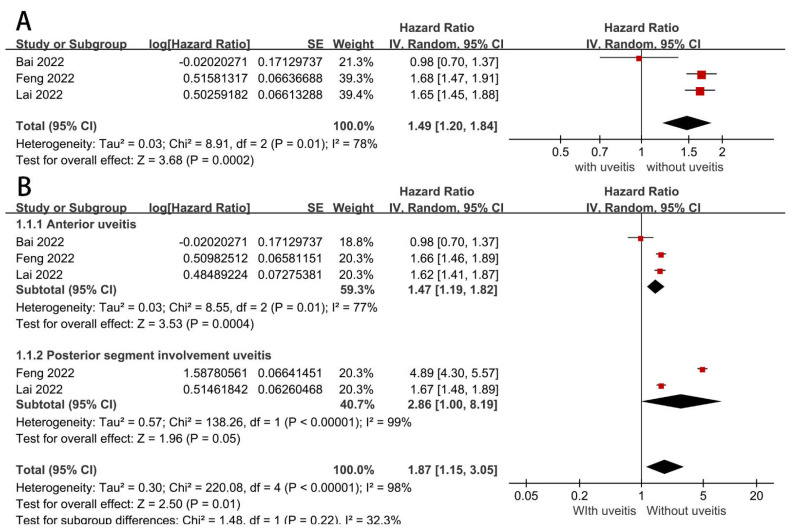
(**A**) Forest plots showing pooled HR with 95% CI of atherosclerosis-related CVD for patients with uveitis versus those without. (**B**) Forest plots showing subgroup uveitis and atherosclerosis-related CVD [27,28,29].

Pooling data from the three cohort studies demonstrated a 1.49-fold increase in the risk of atherosclerosis-related CVD for uveitis patients compared with non-uveitis patients (HR = 1.49, 95% CI = 1.20–1.84, *p* = 0.0002) (Figure 3A). Additionally, significant heterogeneity was presented among individuals (I^2^ = 78%, *p* = 0.01). To identify the potential origin of heterogeneity, leave-one-out sensitivity analysis was conducted. Of these three cohort studies, Bai et al. [27], included AS patients with a history of AAU, whereas the other two studies (Feng et al. [28], and Lai et al. [29]) included AS patients with current combined uveitis. However, excluding Bai et al [27]., did not change the significant difference, but reduced the individual heterogeneity (HR = 1.66, 95% CI = 1.52–1.82, *p* < 0.00001; I^2^ = 0%, *p* = 0.89) (Figure S1), suggesting that significant heterogeneity was caused by the differences in recency of uveitis.

To explore the relationship between the type of uveitis and atherosclerosis-related CVD, we performed a further subgroup analysis. The results showed that anterior uveitis was associated with an increased risk of atherosclerosis-related CVD (HR = 1.47, 95% CI = 1.19–1.82, *p* = 0.0004), with high heterogeneity among studies (I^2^ = 77%, *p* = 0.01). Posterior segment uveitis was also independently associated with atherosclerosis-related CVD (HR = 2.86, 95% CI = 1.00–8.19, *p* = 0.05) and there was significant heterogeneity (I2 = 99%, *p* < 0.00001) (Figure 3B). Visual inspection of funnel plots showed little publication bias (Figure S2).

## 4. Discussion

Our systematic review and meta-analysis first explored the association between uveitis and atherosclerosis-related CVD and indicated that uveitis was not associated with subclinical or clinical carotid atherosclerosis, but with an increased risk of atherosclerosis-related CVD. In particular, acute uveitis significantly increases the risk of atherosclerosis-related CVD by 1.49-fold. Uveitis was a predictor of atherosclerosis-related CVD in new onset of AS patients, even after adjustment for the conventional confounders.

Atherosclerosis is the most common pathological basis for hypertension [30], coronary artery disease [31], peripheral artery disease [32] and cerebrovascular disease [33], and is acknowledged as a predictor of atherosclerosis-related CVD [34,35,36]. Apart from classical atherosclerotic plaques [16,30], subclinical carotid intima-media thickness (cIMT) is also used as a biomarker to assess atherosclerosis. Data from several large studies have supported cIMT as a surrogate marker for the presence and progression of atherosclerosis to assess the risk of CVD [37,38,39,40]. Controversy remains, however, as to whether cIMT can act as a strong independent predictor of CVD. Lorenz et al., published the first meta-analysis evaluating cIMT and cardiovascular events, which showed that increased cIMT was significantly associated with future cardiovascular events, suggesting cIMT as a strong predictor of cardiovascular events [41]. Nevertheless, several subsequent meta-analyses have failed to demonstrate the validity of cIMT in predicting cardiovascular events [42,43,44]. Furthermore, two earlier studies also concluded that the presence of carotid plaques was superior to increased cIMT values in predicting future cardiovascular events [45,46]. The prevailing recommendation is therefore that carotid ultrasound should be used to evaluate both plaque presence and cIMT values in asymptomatic patients at moderate- to high-cardiovascular risk in clinical practice.

As a protective lipoprotein, HDL is able to prevent the development of atherosclerosis by absorbing and removing lipids from plaques and exerting an antioxidant function [47]. Population-based studies have indeed reported associations between high HDL and lower risks of adverse cardiovascular outcomes and mortality [48,49,50]. Under acute and chronic inflammatory conditions, however, HDL can become dysfunctional and act as a pro-inflammatory factor, promoting the accumulation of oxidized low-density lipoprotein (oxLDL) and further enhance the chemotactic activity of monocytes [51]. Accumulated oxLDL can aggravate endothelial dysfunction and promote the development of atherosclerosis by mediating leukocyte activation, the secretion of pro-inflammatory cytokines and the expression of leukocyte adhesion molecules, allowing cell degranulation and the release of reactive oxygen species [52]. Theoretically, in line with other autoimmune inflammatory diseases, including SLE [53,54,55], rheumatoid arthritis [22,56] and Behçet’s disease [57,58], uveitis as an inflammatory disease may lead to the loss of HDL’s antioxidant capacity if prolonged, thereby accelerating atherogenesis and increasing the risk of atherosclerosis-related CVD.

Our meta-analysis reviewed data from three comparisons between patients with uveitis and controls, including two comparisons of cIMT measurements and two comparisons of carotid plaques. Both included articles reported no significant difference in the mean cIMT between uveitis patients or non-uveitis controls, consistent with the findings in the presence of carotid plaques. The pooled data showed no significant difference in the incidence of carotid atherosclerosis between uveitis patients or non-uveitis controls (OR = 1.30, 95% CI = 0.71–2.41, *p* = 0.39). That is, the available data failed to demonstrate an independent association between uveitis and subclinical atherosclerosis. Given the small number of studies reporting data on atherosclerosis in patients with uveitis, and a mean follow-up of less than five years, more studies are needed in the future to investigate whether uveitis is associated with cIMT and carotid plaques.

The three retrospective cohort studies included reported AMI, ACS and MACE, respectively, with a mean follow-up of 3.8 to 9.9 years in the uveitis group versus the non-uveitis group among AS patients. All the included articles reported a significantly higher prevalence of AMI, ACS and MACE in patients with uveitis than in non-uveitis controls, i.e., there was evidence to support that the prevalence of atherosclerosis-related CVD in patients with uveitis was significantly higher than in non-uveitis patients, suggesting that uveitis was independently associated with atherosclerosis-related CVD in AS patients, consistent with published findings in patients with other autoimmune diseases. It is worth noting that these three database-based retrospective studies were all from The National Health Insurance Research Database in Taiwan, which extracted the medical data from new onset AS patients from 2000 to 2015. Thus, further studies in multiple regions in the future should explore whether uveitis is associated with increased atherosclerosis-related CVD.

Several potential limitations of our current study need to be acknowledged. First, the number of studies included in the meta-analysis was small and included a conference abstract that provided limited data. Second, the main population included in the study was adults with AS but another study included children, which may introduce large demographic heterogeneity. Third, the three retrospective cohort studies were not designed with uniform entries for the 4-fold propensity score matching of study cohorts versus comparison cohorts, and not all Cox regression analyses for outcome events used the competing risk model, which may overestimate the risk of developing the endpoint event. Finally, a combination of the above factors led to significant heterogeneity among the included studies, and the small number of studies did not allow for sufficient further subgroup analysis or sensitivity analysis to identify the sources of heterogeneity.

## 5. Further Directions

As a systemic inflammatory state, uveitis is an important predictor of atherosclerosis-associated CVD in patients with new onset AS. Patients with autoimmune diseases combined with uveitis should be given more attention by clinicians for earlier and more careful screening of cardiovascular risk factors and the development of specific cardiovascular prevention strategies. However, three studies of uveitis and atherosclerotic heart disease for which we pooled data were all from the Taiwanese population, and the applicability and generalizability of the findings to other populations remains unclear. Further studies in multiple regions are needed to explore whether uveitis is associated with an increase in atherosclerosis-related CVD. Furthermore, the available data are not yet sufficient to demonstrate an independent association between uveitis and subclinical or clinical atherosclerosis, and more, population-based cohort studies with long follow-up times are needed in the future to clarify the relationship between uveitis, cIMT and carotid plaques.

## 6. Conclusions

In summary, our meta-analysis indicated that uveitis is associated with an increased risk of atherosclerosis-related CVD in patients with AS. Consequently, more attention should be paid to patients with autoimmune diseases complicated with uveitis. Earlier and more careful screening of cardiovascular risk factors and more specific cardiovascular prevention strategies may be associated with a better prognosis.

## Figures and Tables

**Table 1:** Characteristics of the included studies.

Study (First Author-Year)	Study Design	Regions and Number of Centers	Duration of Study	Participants	Sample. *n*	Male (%)	Uveitis (%)	Without Uveitis (%)	Definition of Uveitis	Type of Uveitis	Main Cardiovascular Outcomes	Follow-Up	Quality Score
Bai 2022 [27]	Retrospective cohort study	Taiwan (The Longitudinal Health Insurance Database)	1 January 2003–31 December 2013	Newly diagnosed AS patients	17,720	11,535 (65.1)	3544 (20.0)	14176 (80.0)	ICD-9-CM codes	History of AAU	MACE *	AAU: 3.8 ± 1.9 years, Non-AAU: 3.9 ± 1.8 years	7
Berg 2014	Cross-sectional study	Norway (one-center)	2008–2009	AS patients	159	98 (61.6)	84 (52.8)	75 (47.2)	NA	History of uveitis	Hypertension, atherosclerosis	NA	6
Conkar 2017	Cross-sectional study	Turkey (one-center)	1 January 2010–1 March 2017	Children	125	55 (44.0)	75 (60.0)	50 (40.0)	Chronic uveitis: Persistent and characterized by prompt relapse (in <3 months) after discontinuation of therapy	Intermediate uveitis 58.7%, anterior uveitis 33.3%, panuveitis 8%	Subclinical atherosclerosis	3.2 ± 3.04 years	9
Feng 2022 [28]	Retrospective cohort study	Taiwan (The Longitudinal Health Insurance Database)	1 January 2000–31 December 2015	New onset of AS patients	5555	3105 (55.9)	1111 (20.0)	4444 (80.0)	ICD-9-CM codes	Anterior uveitis (35.5%), posterior segment involvement uveitis (64.5%)	ACS	Uveitis: 9.82 ± 8.40 years, Without uveitis: 9.85 ± 8.55 years	9
Gonzalez Mazon 2021	Cross-sectional study	Spain (Multi-center)	NA	Axial spondylarthritis patients	886	599 (67.6)	177 (20.0)	709 (80.0)	NA	Anterior uveitis	Carotid subclinical atherosclerosis	NA	4
Lai 2022 [29]	Retrospective cohort study	Taiwan (The National Health Insurance Research Database)	January 2000–December 2015	New onset of AS patients	5905	3255 (55.1)	1181 (20.0)	4724 (80.0)	ICD-9-CM codes	Anterior uveitis (36.6%), posterior segment uveitis (63.4%)	AMI	9.91 ± 8.56 years	9

* MACE indicates those who have been hospitalized for at least 3 days (unless patients died) with a diagnosis of myocardial infarction receiving a coronary artery bypass graft or percutaneous coronary intervention/percutaneous transluminal coronary angioplasty/stent, or with a hospital discharge diagnosis of ischemic stroke. AS: ankylosing spondylitis, AAU: acute anterior uveitis.

**Table 2:** Types of uveitis and adjusted HRs on atherosclerosis-related CVD included in the study.

Study (First Author-Year)	With Uveitis	Anterior Uveitis	Posterior Segment Involvement Uveitis
Bai 2022 [27]	NA	0.980 (0.700–1.370)	NA
Feng 2022 [28]	1.675 (1.501–1.947)	1.665 (1.495–1.935)	4.893 (4.385–5.689)
Lai 2022 [29]	1.653 (1.480–1.918)	1.624 (1.409–1.874)	1.673 (1.510–1.930)

## Data Availability

Not applicable.

## Supplementary Materials

The following supporting information can be downloaded at: https://www.mdpi.com/article/10.3390/diagnostics12123178/s1.
