# Supplementary material for: Association Between Atherosclerosis-Related Cardiovascular Disease and Uveitis: A Systematic Review and Meta-Analysis
Source: Diagnostics (Basel). 2022 Dec 15;12(12):3178. doi: 10.3390/diagnostics12123178 (PMC9777442; doi:10.3390/diagnostics12123178)
Supplement: Supplementary file 1 [file diagnostics-12-03178-s001.zip › diagnostics-2024729-supplementary.pdf]

Supplementary Material

Study or Subgroup	log[Hazard Ratio]	SE	Weight	Hazard Ratio IV, Random, 95% CI
Bai 2022	-0.02020271	0.17129737	0.0%	0.98 [0.70, 1.37]
Feng 2022	0.51581317	0.06636688	49.8%	1.68 [1.47, 1.91]
Lai 2022	0.50259182	0.06613288	50.2%	1.65 [1.45, 1.88]
**Total (95% CI)**			**100.0%**	**1.66 [1.52, 1.82]**

Heterogeneity: Tau² = 0.00; Chi² = 0.02, df = 1 (P = 0.89); I² = 0%

Test for overall effect: Z = 10.87 (P < 0.00001)

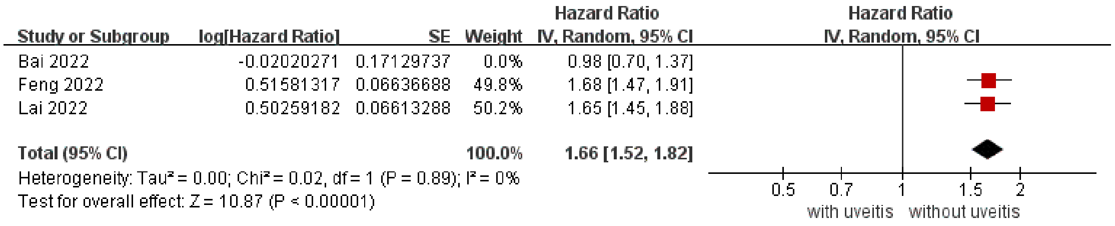

Figure S1. Sensitive analysis of atherosclerosis-related CVD for the patients with uveitis versus those without [27–29].

Supplementary Table S1. Quality assessment of the cohort studies using the Newcastle Ottawa scale (NOS).

Author/Year of publication	Selection	Comparability	Outcome	Final score
**Lai-2022 [29]**	****	**	***	9
**Feng-2022 [28]**	****	**	***	9
**Bai-2022 [27]**	***	**	**	7

Supplementary Table S2. Quality assessment of the cross-sectional studies using the American Agency for Healthcare Research and Quality (AHRQ) criterion.

Author/Year of publication	Item 1	Item 2	Item 3	Item 4	Item 5	Item 6	Item 7	Item 8	Item 9	Item 10	Item 11	Final score
**Berg-2014**	1	0	1	1	0	1	0	1	0	0	0	5
**Conkar-2017**	1	1	1	1	0	1	1	1	0	0	1	8
**Gonzalez Mazon-2021**	1	0	0	0	0	1	0	1	0	0	0	3

Supplementary Table S3. Multivariate variables adjusted for estimates of HR in included cohort studies.

Author/Year of publication	Multivariate variables
**Lai-2022 [29]**	Uveitis, age, sex, DM, hyperlipidemia, hypertension, cerebrovascular accident, CHF, COPD, asthma, CAD, cardiomegaly, metabolic syndrome, Charlson comorbidity index
**Feng-2022 [28]**	Age, sex, DM, hyperlipidemia, hypertension, COPD, asthma, systemic steroids
**Bai-2022 [27]**	AAU, sex, urbanization, low income, length of hospital stays, HF, hypertension, DM, hyperlipidemia, ischemic heart disease, ischemic stroke, pulmonary disease, CKD, chronic liver disease, IBD, psoriasis, AS treatment with NSAID, methotrexate, sulfasalazine, steroid

DM: diabetes mellitus; CHF: congestive heart failure; COPD: chronic obstructive pulmonary disease; CAD: coronary artery disease; AAU: acute anterior uveitis; HF: heart failure; CKD: chronic kidney disease; IBD: inflammatory bowel disease; AS: ankylosing spondylitis; NSAID: nonsteroidal antiinflammatory drug
